# Supplementary material for: Biophysical characterization of Eag chaperones suggests the mechanism of effector transmembrane domain release
Source: Nat Commun. 2026 Jan 22;17:1401. doi: 10.1038/s41467-025-68138-w (PMC12881460; doi:10.1038/s41467-025-68138-w)
Supplement: Supplementary file 1 — Supplementary Information [file 41467_2025_68138_MOESM1_ESM.pdf]

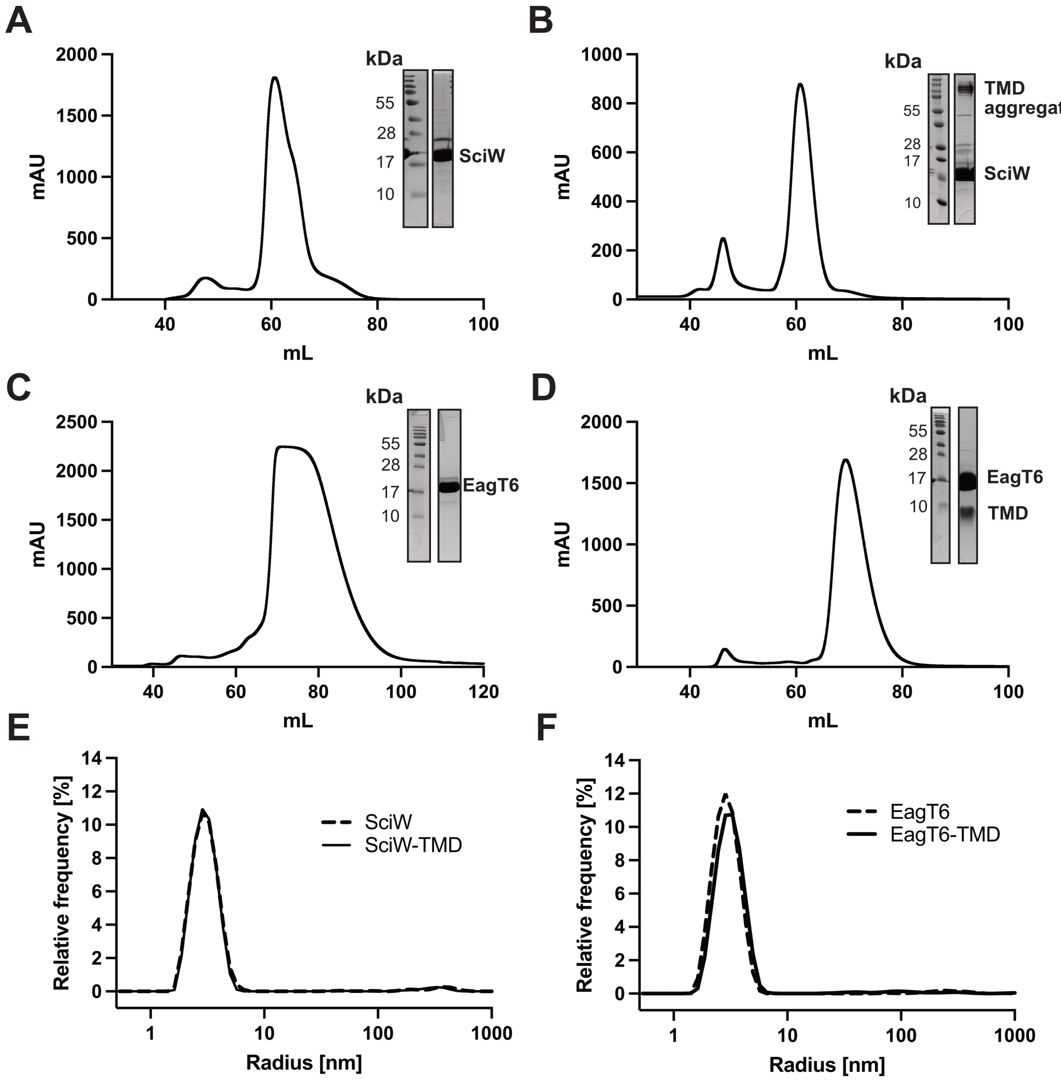

Supplementary Figure 1: Purification of Eag and Eag-TMD complexes. (A) SciW and **(B)** SciW-TMD **(C)** EagT6 and **(D)** EagT6-TMD. All panels show the final purified material after size-exclusion chromatography (SEC) using a HiLoad 16/600 Superdex75 preparatory grade column. The final SEC buffer was 50 mM Tris pH 8.0, 250 mM NaCl, 1 mM BME. To confirm purity, fractions were run on a 13% SDS-PAGE gel and visualized with Coomassie Brilliant Blue. Eag chaperones and TMD bands are marked on each gel. Dynamic light scattering (DLS) measured intensity distributions versus radius of wild-type **(E)** SciW and SciW-TMD and **(F)** EagT6 and EagT6-TMD. All measurements were collected in 50 mM Tris pH 8.0, 250 mM NaCl, and performed at room temperature at a concentration of 0.5 mg/mL in triplicate. Particle sizes are shown in Supplementary Table 2.

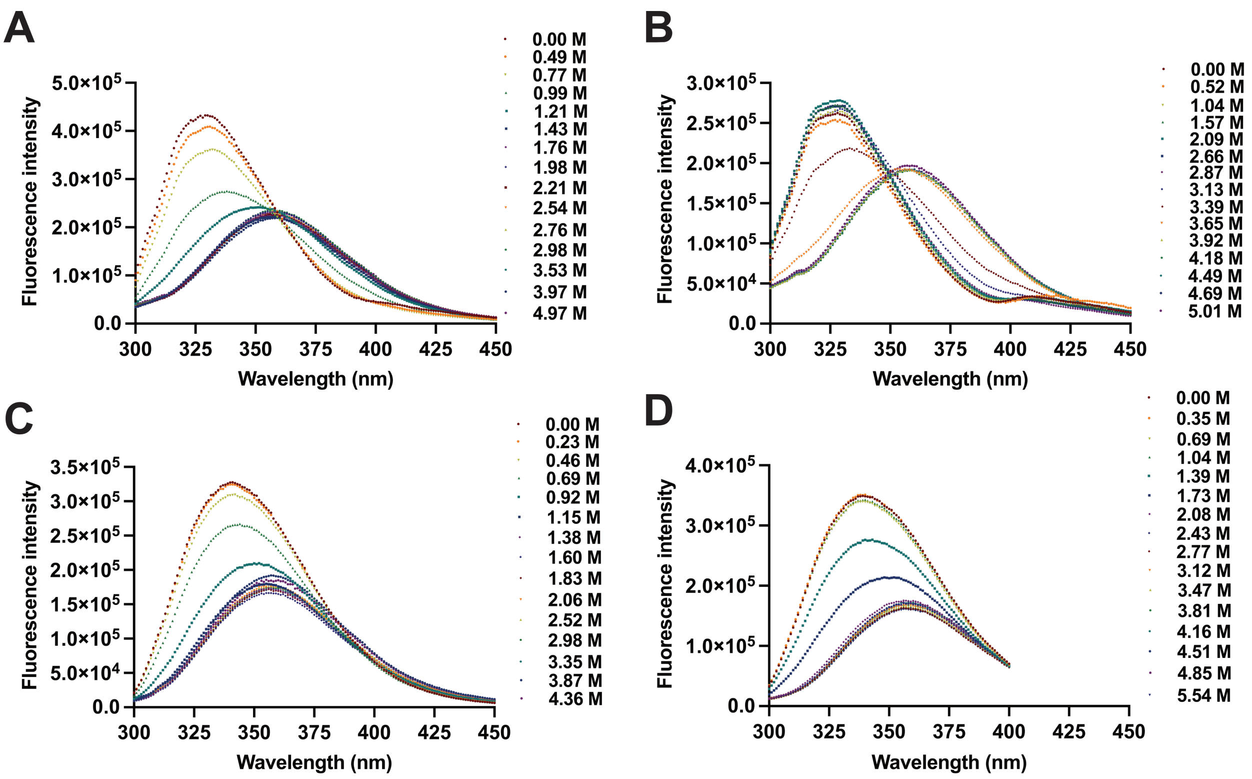

Supplementary Figure 2: Steady-state fluorescence spectrum of Eag and Eag-TMD complexes in guanidine hydrochloride (GdmCl). Steady-state fluorescence spectrum of **(A)** SciW and **(B)** SciW-TMD and **(C)** EagT6 and **(D)** EagT6-TMD collected in 50 mM Tris pH 8.0, 250mM NaCl in increasing concentrations of GdmCl. All measurements were performed at room temperature 293 K with a stock protein concentration of 10 µM diluted to a final concentration of 1 µM. The samples were excited at 280 nM with excitation and emission slits set at 2 nM bandpass.

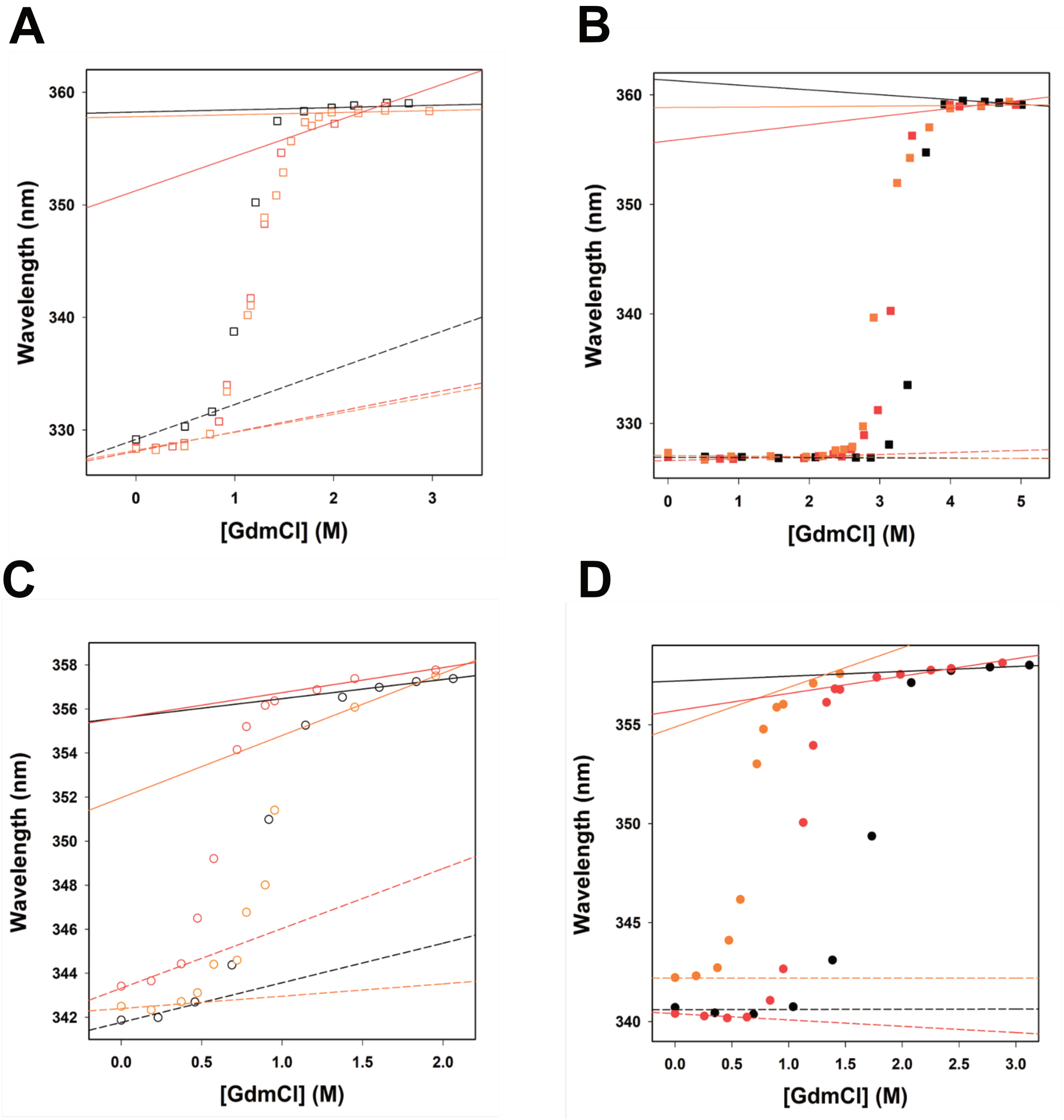

E

SciW-APO	λ_{folded}	$\lambda_{unfolded}$	SciW-TMD	λ_{folded}	$\lambda_{unfolded}$
WT	328.2+1.6[GdmCl]	358.2+0.2[GdmCl]	WT	326.9-0.0[GdmCl]	361.3-0.4[GdmCl]
L66A	328.1+1.7[GdmCl]	358.8+0.2[GdmCl]	L66A	327.1-0.1[GdmCl]	355.8+0.7[GdmCl]
Q58A	328.3-0.5[GdmCl]	355.0+1.4[GdmCl]	Q58A	326.6+0.2[GdmCl]	358.9+0.1[GdmCl]
EagT6-APO			EagT6-TMD		
WT	341.7+1.8[GdmCl]	355.6+0.9[GdmCl]	WT	340.6+0.0[GdmCl]	358.2+0.3[GdmCl]
L66A	343.3+2.7[GdmCl]	355.6+1.4[GdmCl]	L66A	340.4-0.3[GdmCl]	355.7+0.9[GdmCl]
Q58A	342.4+0.6[GdmCl]	352.0+2.8[GdmCl]	Q58A	342.2+0.0[GdmCl]	354.6+2.0[GdmCl]

Supplementary Figure 3: Fraction unfolding fits from steady-state fluorescence data. Unfolding profile of Eag chaperones, SciW (Squares, panels A and B) and EagT6 (circles, panels C and D) as represented by the lambda max *λmax* of the fluorescence spectra. [Empty shapes = APO, filled shapes = TMD bound, WT is represented in black,

Q58A mutant is represented in orange, L66A mutant is represented in red]. It can be clearly seen that these unfolding profiles exhibit three distinct regimes: 1) an initial regime that is linearly dependent on denaturant concentration and represents how the maximum of the folded protein λf changes with denaturant concertation; 2) a nonlinear transition regime indicating the protein unfolding process; 3) a final regime that is also linearly dependent upon denaturation concentration, showing how the maximum of the unfolded protein λu changes with denaturant concentration. (E) Table listing the pre- and post-transition slopes shown in the figure.

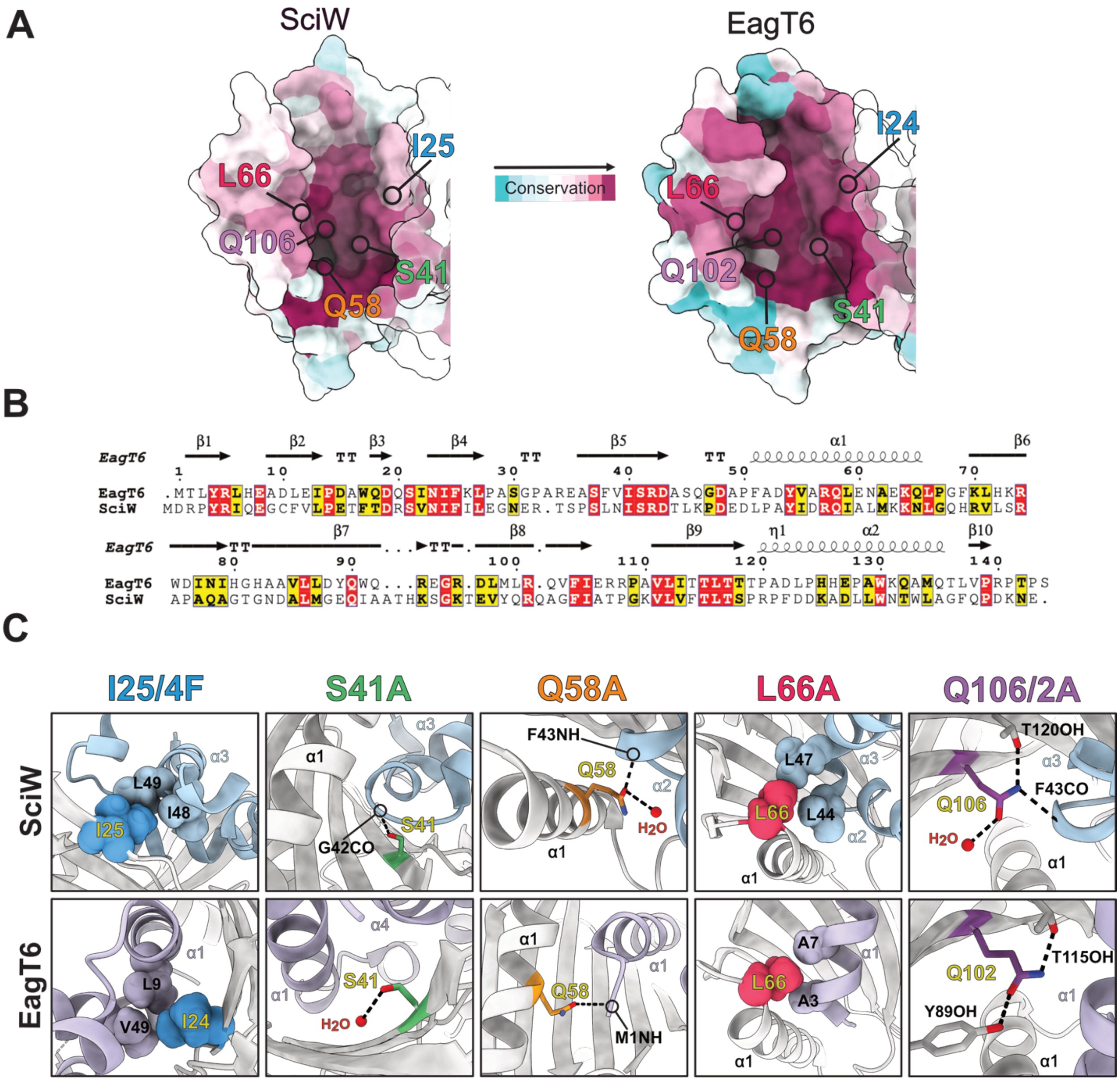

Supplementary Figure 4: Conservation and critical residues involved in Eag-TMD binding. Surface conservation representation of the TMD binding pocket in each chaperone monomer **(A)** SciW (left) and EagT6 (right). Conserved residues targeted for point mutation are indicated. Multisequence alignment of SciW and EagT6 as calculated by Consurf (https://consurf.tau.ac.il/). Molecular graphics were drawn using USCF ChimeraX (https://www.rbvi.ucsf.edu/chimerax/). **(B)** Multisequence alignment of SciW and EagT6 as plotted by Espript3 (https://espript.ibcp.fr/). **(C)** Molecular details of point variant residues involved in binding the TMD in opposing chain shown in Figure 3. SciW

(top row) and EagT6 (bottom row) are drawn. Residues are substituted to alanine and colored. I24/I25F (blue) creates a hydrophobic surface in the palm of the claw. Hydrophilic residues S41A (green) and Q58A (orange) form bifurcated hydrogen bonds with the TMD backbone. L66A (red) provides a “knob” for the hole of its cognate TMD. Q106/2A (purple) form bifurcated hydrogen bonds with the TMD backbone.

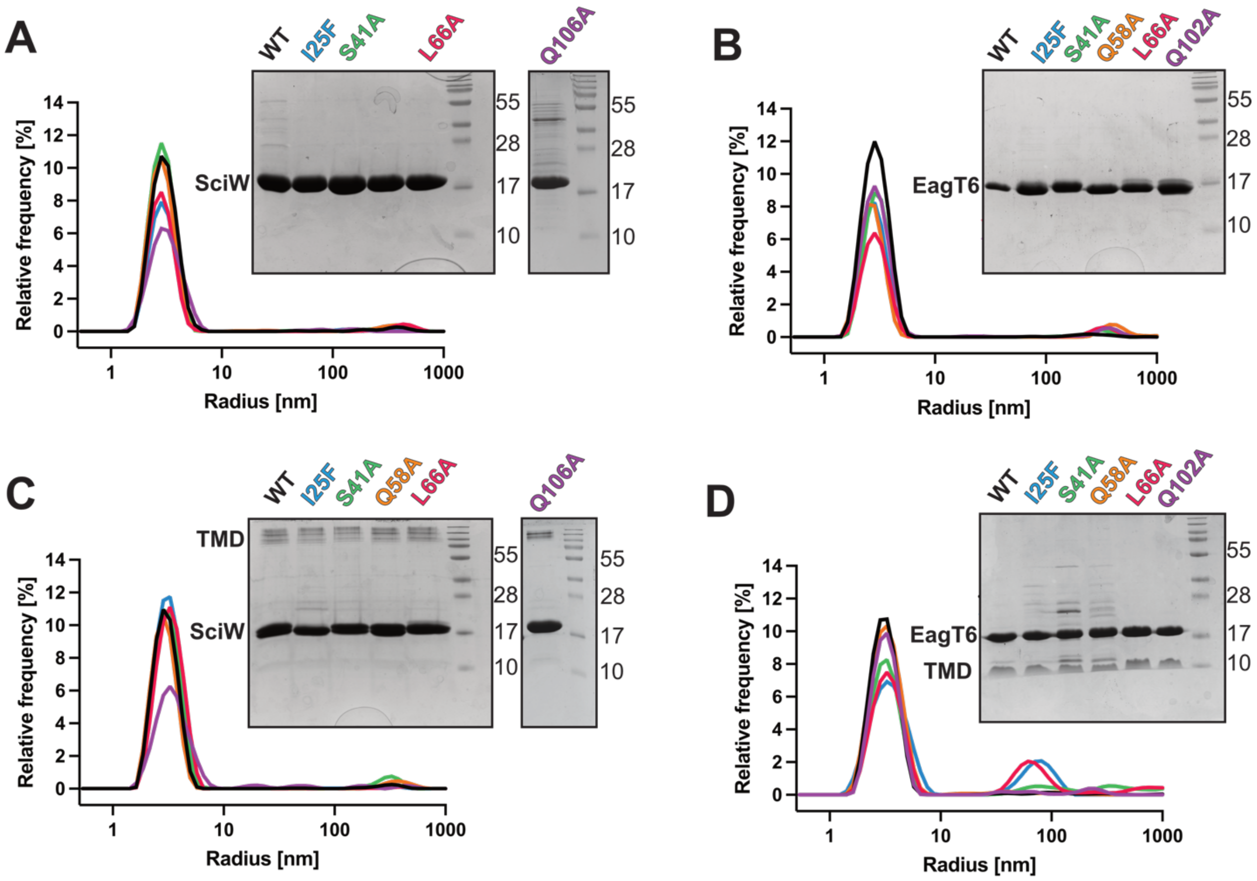

Supplementary Figure 5: Eag and Eag:TMD point variants are properly folded and bind effector TMDs. DLS measured intensity distributions versus radius for **(A)** SciW, **(B)** EagT6, **(C)** SciW-TMD and **(D)** EagT6-TMD. All measurements were collected in 50mM Tris pH 8.0, 250mM NaCl and performed at room temperature at a concentration of 0.5 mg/mL in triplicate. Each panel also shows a Coomassie stained SDS-PAGE gel for each purified variant as compared to the wild-type. Molecular weight markers are in kDa.

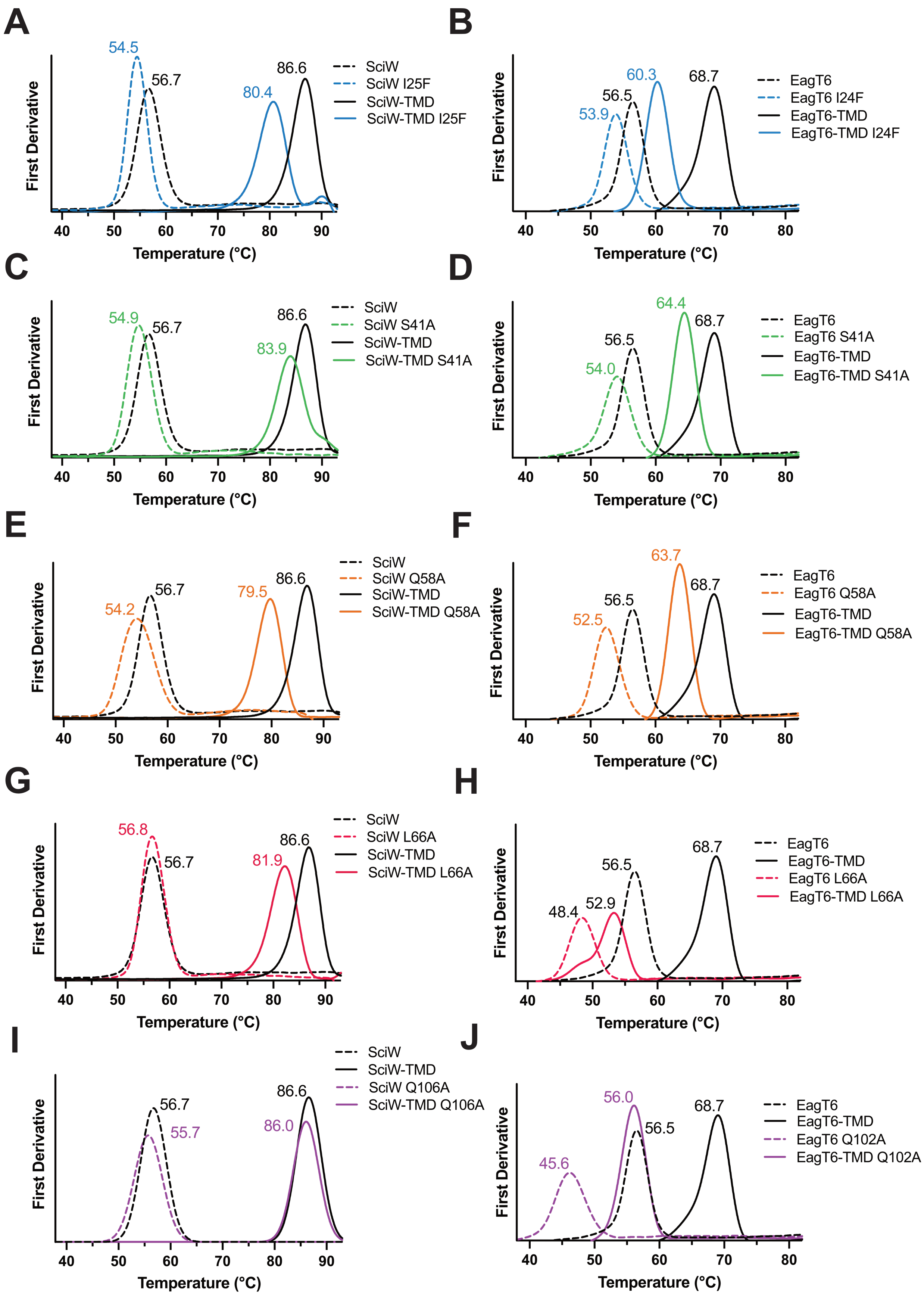
A
54.5
56.7
80.4
86.6
SciW
SciW I25F
SciW-TMD
SciW-TMD I25F
First Derivative
Temperature (°C)
B
53.9
56.5
60.3
68.7
EagT6
EagT6 I24F
EagT6-TMD
EagT6-TMD I24F
First Derivative
Temperature (°C)
C
54.9
56.7
83.9
86.6
SciW
SciW S41A
SciW-TMD
SciW-TMD S41A
First Derivative
Temperature (°C)
D
54.0
56.5
64.4
68.7
EagT6
EagT6 S41A
EagT6-TMD
EagT6-TMD S41A
First Derivative
Temperature (°C)
E
54.2
56.7
79.5
86.6
SciW
SciW Q58A
SciW-TMD
SciW-TMD Q58A
First Derivative
Temperature (°C)
F
52.5
56.5
63.7
68.7
EagT6
EagT6 Q58A
EagT6-TMD
EagT6-TMD Q58A
First Derivative
Temperature (°C)
G
56.8
56.7
81.9
86.6
SciW
SciW L66A
SciW-TMD
SciW-TMD L66A
First Derivative
Temperature (°C)
H
48.4
52.9
56.5
68.7
EagT6
EagT6-TMD
EagT6 L66A
EagT6-TMD L66A
First Derivative
Temperature (°C)
I
56.7
55.7
86.6
86.0
SciW
SciW-TMD
SciW Q106A
SciW-TMD Q106A
First Derivative
Temperature (°C)
J
45.6
56.0
56.5
68.7
EagT6
EagT6-TMD
EagT6 Q102A
EagT6-TMD Q102A
First Derivative
Temperature (°C)

Supplementary Figure 6: Thermal denaturation of Eag and Eag-TMD point variants measured by nanoDSF. Each panel shows an Eag and Eag-TMD point variant relative to the wild-type protein (black). SciW point-variants (left side) **A, C, E, G, I** and EagT6 point variants (right side) are panels **B, D, F, H, J**. Apo Eag chaperones (unbound) are represented by dashed lines and Eag-TMD complexes by soldi lines. Eag point variants are coloured as follows: I25/4F (blue), S41A (green), Q58A (orange), L66A, (red), Q106/2A (purple), and wild-type (black). All measurements were performed in 50 mM Tris pH 8.0, 250 mM NaCl, 1 mM BME at 25 °C at a concentration of 0.5 mg/mL.

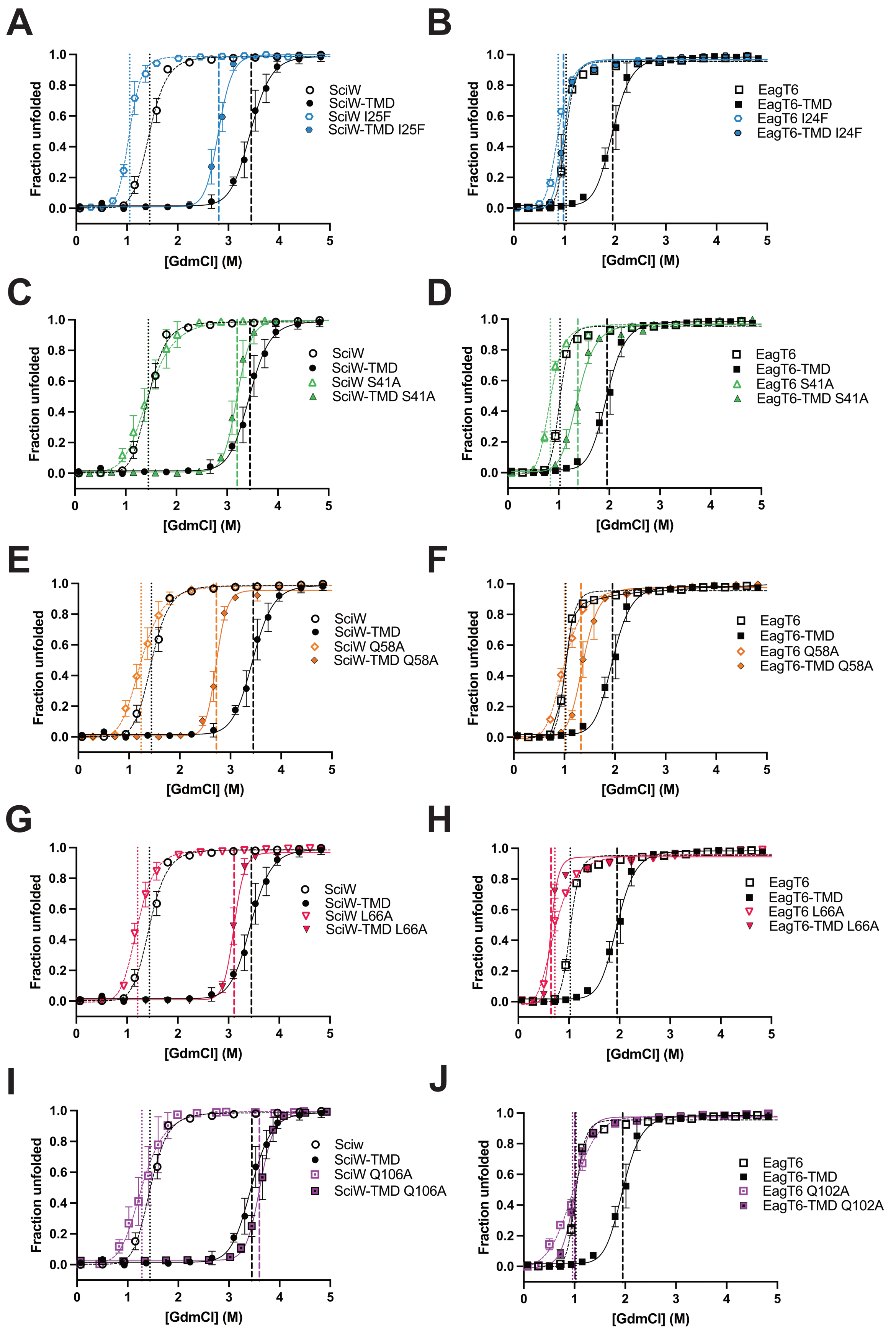
A
B
C
D
E
F
G
H
I
J
Fraction unfolded
[GdmCl] (M)
SciW
SciW-TMD
SciW I25F
SciW-TMD I25F
EagT6
EagT6-TMD
EagT6 I24F
EagT6-TMD I24F
SciW S41A
SciW-TMD S41A
EagT6 S41A
EagT6-TMD S41A
SciW Q58A
SciW-TMD Q58A
EagT6 Q58A
EagT6-TMD Q58A
SciW L66A
SciW-TMD L66A
EagT6 L66A
EagT6-TMD L66A
Sciw
SciW Q106A
SciW-TMD Q106A
EagT6 Q102A
EagT6-TMD Q102A

Supplementary Figure 7: **Chemical denaturation of Eag and Eag:TMD point variants measured by nanoDSF.** Each panel shows an Eag or Eag-TMD point variant relative to the wild-type protein (black) subjected to increasing concentrations of guanidine hydrochloride (GdmCl). SciW point variants (left side) are panels **A, C, E, G, I** and EagT6 point variants (right side) are panels **B, D, F, H, J**. Apo Eag chaperones (unbound) are represented by unfilled shapes and Eag-TMD complexes by filled shapes. Eag point variants are coloured as follows: I25/4F (blue), S41A (green), Q58A (orange), L66A (red), Q106/2A (purple), wild-type (black). Vertical lines indicate inflection point (C_{50} [M]) and are colored by variant. Error bars represent the standard deviation of three replicates.

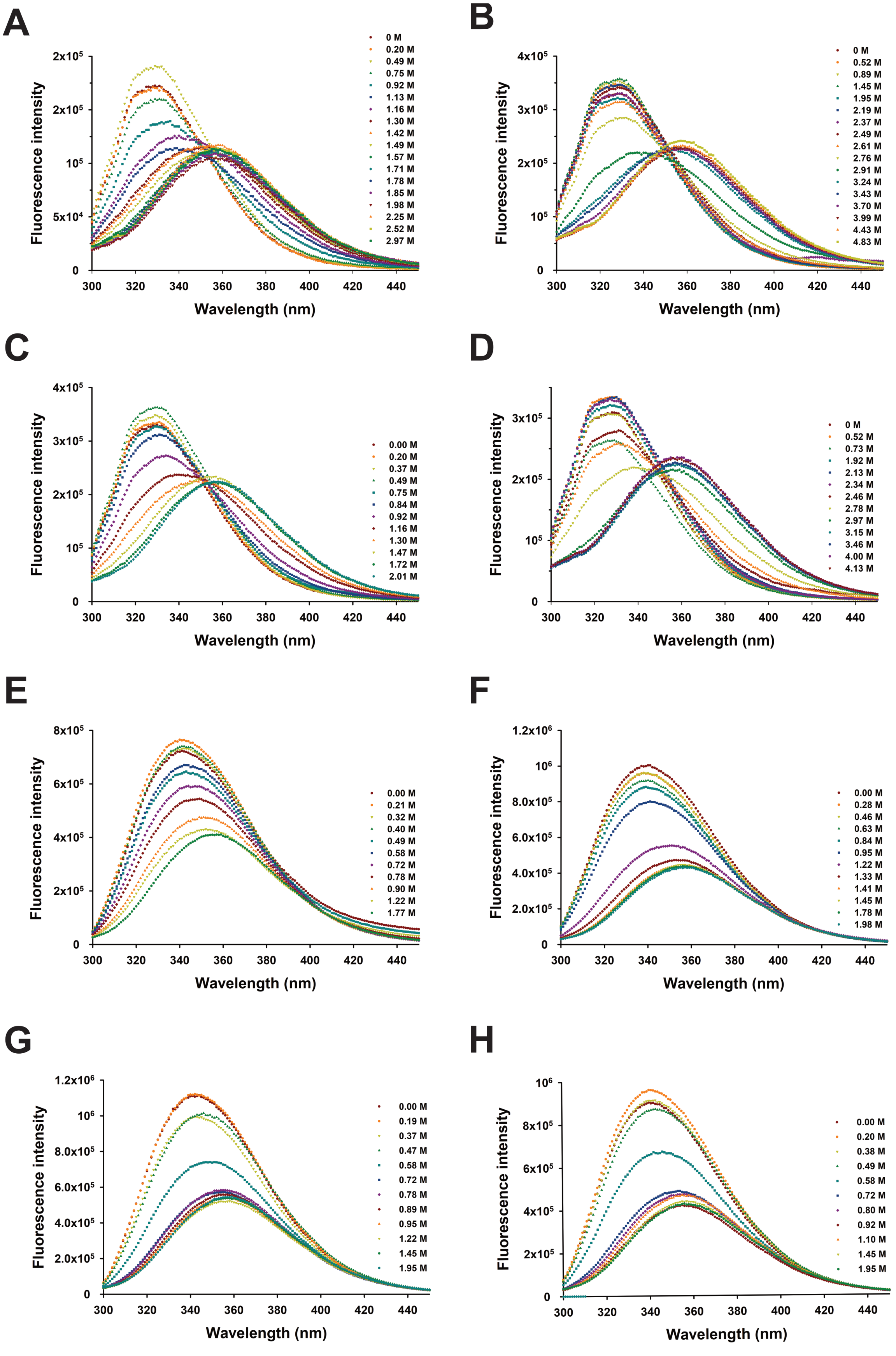

A
Fluorescence intensity
Wavelength (nm)
0 M
0.20 M
0.49 M
0.75 M
0.92 M
1.13 M
1.16 M
1.30 M
1.42 M
1.49 M
1.57 M
1.71 M
1.78 M
1.85 M
1.98 M
2.25 M
2.52 M
2.97 M
B
Fluorescence intensity
Wavelength (nm)
0 M
0.52 M
0.89 M
1.45 M
1.95 M
2.19 M
2.37 M
2.49 M
2.61 M
2.76 M
2.91 M
3.24 M
3.43 M
3.70 M
3.99 M
4.43 M
4.83 M
C
Fluorescence intensity
Wavelength (nm)
0.00 M
0.20 M
0.37 M
0.49 M
0.75 M
0.84 M
0.92 M
1.16 M
1.30 M
1.47 M
1.72 M
2.01 M
D
Fluorescence intensity
Wavelength (nm)
0 M
0.52 M
0.73 M
1.92 M
2.13 M
2.34 M
2.46 M
2.78 M
2.97 M
3.15 M
3.46 M
4.00 M
4.13 M
E
Fluorescence intensity
Wavelength (nm)
0.00 M
0.21 M
0.32 M
0.40 M
0.49 M
0.58 M
0.72 M
0.78 M
0.90 M
1.22 M
1.77 M
F
Fluorescence intensity
Wavelength (nm)
0.00 M
0.28 M
0.46 M
0.63 M
0.84 M
0.95 M
1.22 M
1.33 M
1.41 M
1.45 M
1.78 M
1.98 M
G
Fluorescence intensity
Wavelength (nm)
0.00 M
0.19 M
0.37 M
0.47 M
0.58 M
0.72 M
0.78 M
0.89 M
0.95 M
1.22 M
1.45 M
1.95 M
H
Fluorescence intensity
Wavelength (nm)
0.00 M
0.20 M
0.38 M
0.49 M
0.58 M
0.72 M
0.80 M
0.92 M
1.10 M
1.45 M
1.95 M

Supplementary Figure 8: Steady-state fluorescence spectrum of apo Eag and Eag-TMD variants Q58A and L66A. Steady-state fluorescence spectrum of **(A)** SciW Q58A **(B)** SciW-TMD Q58A **(C)** SciW L66A **(D)** SciW-TMD L66A **(E)** EagT6 Q58A **(F)** EagT6-TMD Q58A **(G)** EagT6 L66A and **(H)** EagT6-TMD L66A collected in 50 mM Tris (pH 8.0), 250mM NaCl in increasing concentrations of guanidine hydrochloride (GdmCl). All measurements were performed at room temperature 293 K with a stock protein concentration of 10 μM diluted to a final concentration of 1 μM. The samples were excited at 280 nM with excitation and emission slits set at 2 nM bandpass.

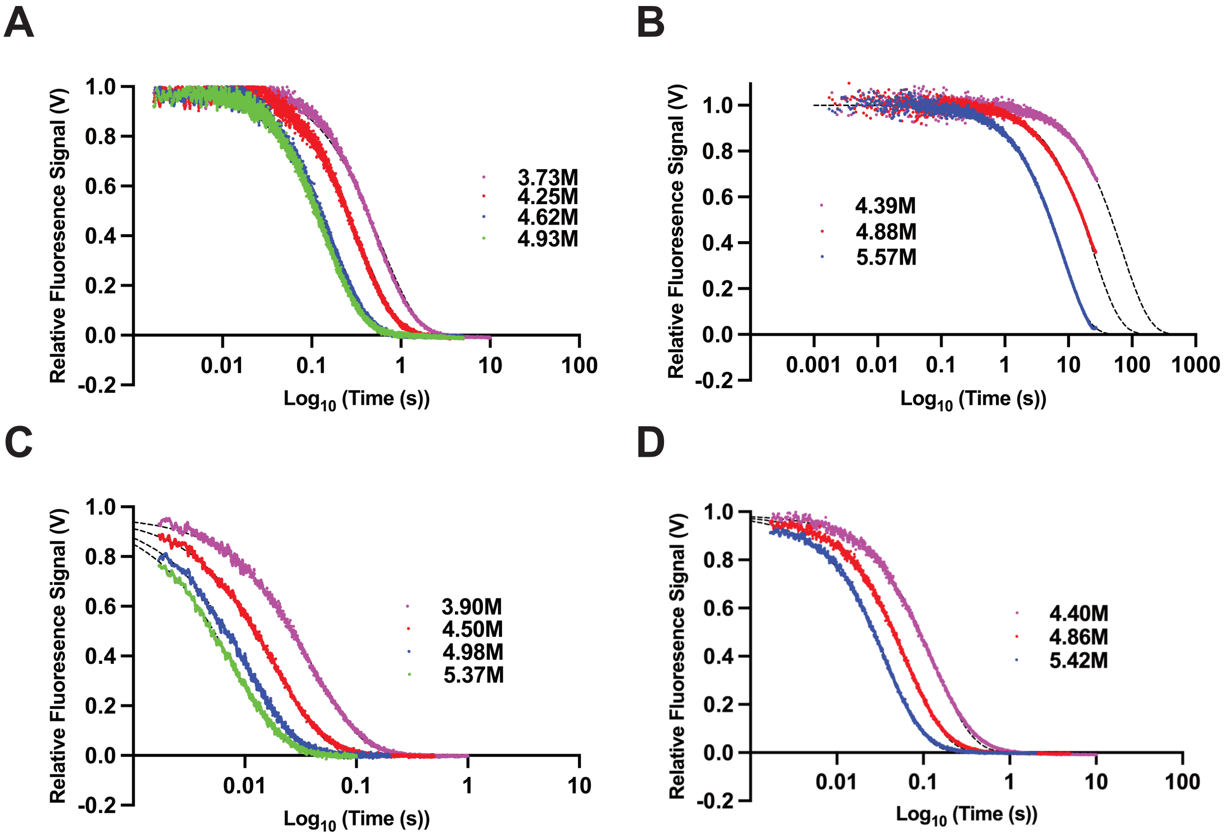

Supplementary Figure 9: Fluorescent data for wild-type Eag and Eag:TMD wild-type complex unfolding kinetics Stopped-flow kinetics traces of **(A)** SciW, **(B)** SciW-TMD **(C)** EagT6, and **(D)** EagT6:TMD in 50 mM Tris (pH 8.0), 250mM NaCl at 20°C. Unfolding traces (fluorescence decrease) are measured in the increasing guanidine hydrochloride (GdmCl) concentrations listed. The change in protein fluoresce is monitored at 330 nm and is normalized to 1. The dashed lines represent the best mono-exponential fits to the obtained data.

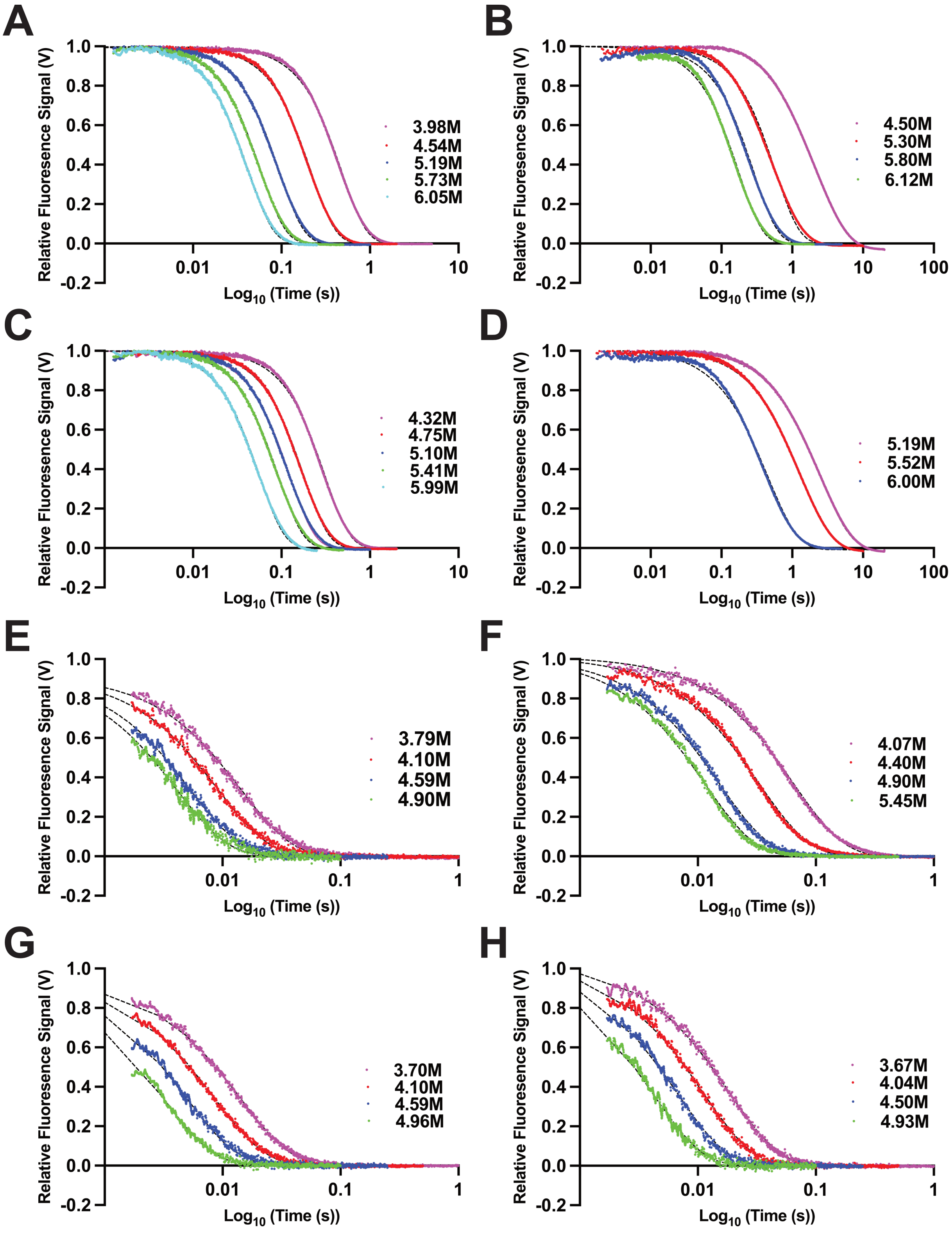
A
B
C
D
E
F
G
H
Relative Fluoresence Signal (V)
Log10 (Time (s))
3.98M
4.54M
5.19M
5.73M
6.05M
4.50M
5.30M
5.80M
6.12M
4.32M
4.75M
5.10M
5.41M
5.99M
5.19M
5.52M
6.00M
3.79M
4.10M
4.59M
4.90M
4.07M
4.40M
4.90M
5.45M
3.70M
4.10M
4.59M
4.96M
3.67M
4.04M
4.50M
4.93M

Supplementary Figure 10: Fluorescent data for of Eag and Eag:TMD complex point variant unfolding kinetics Stopped-flow kinetics traces of **(A)** SciW Q58A **(B)** SciW:TMD Q58A, **(C)** SciW L66A, **(D)** SciW:TMD L66A, **(E)** EagT6 Q58A, **(F)** EagT6:TMD Q58A, **(G)** EagT6 L66A, **(H)** EagT6:TMD L66A in 50 mM Tris (pH 8.0), 250mM NaCl at 20°C. Unfolding traces (fluorescence decrease) are measured in increasing guanidine hydrochloride (GdmCl) concentrations listed. The change in protein fluoresce is monitored at 330 nm and is normalized to 1. The dashed lines represent the best mono-exponential fits to the obtained data.

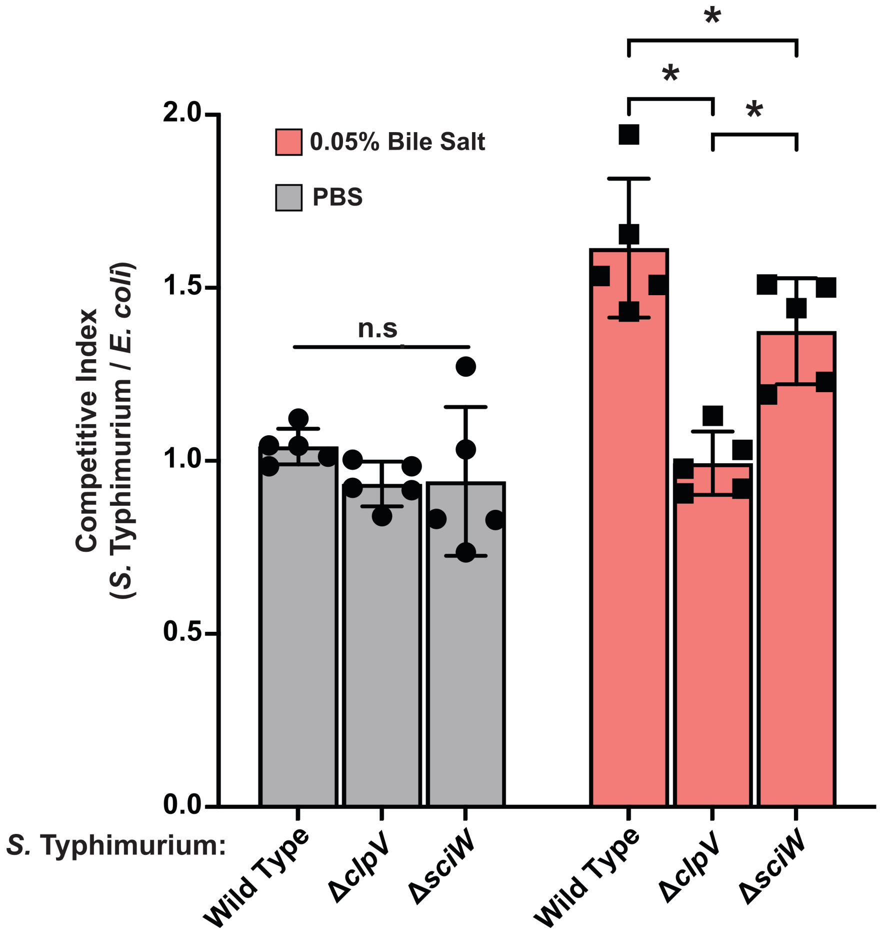

Supplementary Figure 11: Interbacterial Competition Assays between *Salmonella* Typhimurium SL1344 and *E. coli* DH5α. Wild-type and mutants *ΔclpV* (*SL1344_0266*) and *ΔsciW* (*SL1344_0285*) were mixed with *E. coli* at a 1:1 ration and incubated for 48hrs on LB agar plates supplement with 0.05% bile salts or PBS (uninduced SPI-6 T6SS). Each bar represents the average of independent competition assays (n = 5 biological replicates). Error bars represent standard deviation, and statistical significance is indicated by * ($p < 0.05$) or n.s. = not significant. For wild-type and *ΔclpV* + bile salts p = 0.0002, for *ΔsciW* and wild-type + bile salts p = 0.035, for *ΔsciW* and *ΔclpV* + bile salts p = 0.004. Note that *clpV* is *STM0272* and *sciW* is *STM0290* in strain LT2.

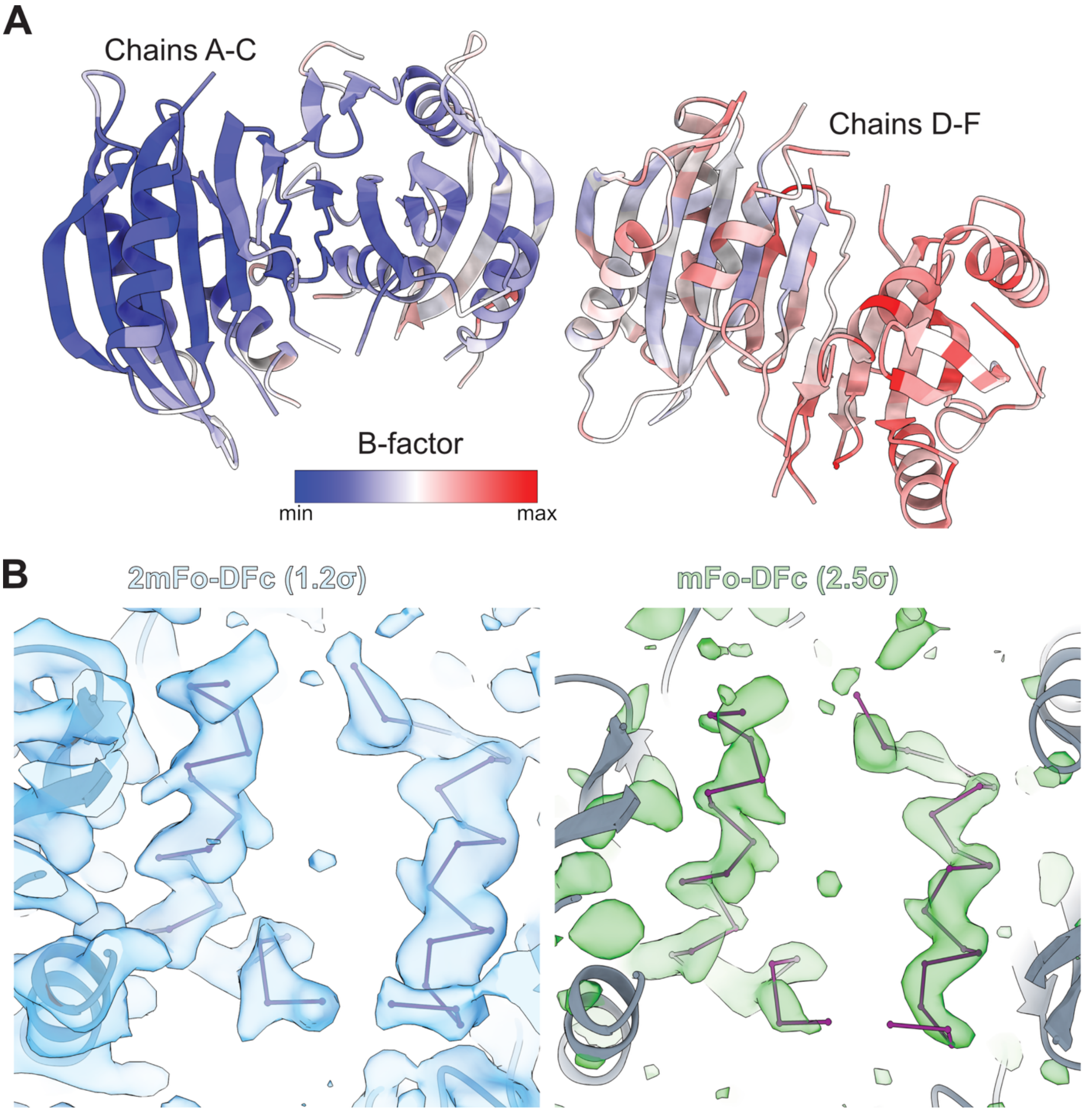

Supplementary Figure 12: SciW-TMD L66A structural modeling data. (A) Asymmetric unit contents colored by B-factor from lowest (blue) to highest (red). **(B)** Electron density maps for the bound TMD in Chains A-C. Right: 2mFo-DFc maps contoured at 1.2 rmsd (~1.2σ) shown in blue. Left: mFo-DFc difference maps contoured at 2.5 rmsd (~2.5σ). Green is positive density. The mFo-DFc map was generated after first omitting chain C (Rhs1 TMD) before refinement. Maps were drawn using ChimeraX.

Supplementary Table 1: NanoDSF thermal and steady-state fluorescent chemical denaturation values and fit paraments of wild-type Eag and Eag-TMD complexes.

Protein	Tm			Urea		GdmCl	
	°C	Δ°C	R^2	C_{50} [M]	R^2	C_{50} [M]	R^2
**SciW**	56.7	30.5	0.917	3.41	0.991	1.45	0.995
**SciW-TMD**	86.6		0.988	N/A	N/A	3.45	0.984
**EagT6**	56.5	12.2	0.947	3.34	0.997	1.03	0.993
**EagT6-TMD**	68.7		0.970	5.89	0.943	2.03	0.976

The melting temperature (Tm) was fit to a gaussian distribution and coefficients of determination (R^2) provided. The Δ°C represents the difference in Tm between the apo and TMD-bound chaperone complexes. The urea and guanidine hydrochloride (GdmCl) denaturation's were fit to a variable slope model and subsequent C_{50} [M] values provided. The R^2 values are > 0.94 suggesting the unfolding of Eag and Eag-TMD complexes are well-described by a variable slope model using the Prometheus NT.48 software and GraphPad Prism.

Supplementary Table 2: Dynamic light scattering (DLS) parameters of Eag and Eag-TMD point variants.

Variant	Protein	Hydrodynamic radius (nm)	Polydispersity Index
**Wild-type**	SciW	2.91 ± 0.03	0.17 ± 0.03
	SciW-TMD	3.04 ± 0.09	0.12 ± 0.02
	EagT6	2.97 ± 0.03	0.14 ± 0.01
	EagT6-TMD	3.18 ± 0.01	0.16 ± 0.01
**I25/4F**	SciW I25F	2.99 ± 0.03	0.13 ± 0.01
	SciW-TMD I25F	3.21 ± 0.09	0.16 ± 0.02
	EagT6 I24F	2.82 ± 0.16	0.12 ± 0.03
	EagT6-TMD I24F	3.43 ± 0.07	0.23 ± 0.06
**S41A**	SciW S41A	2.96 ± 0.08	0.13 ± 0.01
	SciW-TMD S41A	3.16 ± 0.07	0.13 ± 0.03
	EagT6 S41A	3.05 ± 0.03	0.13 ± 0.02
	EagT6-TMD S41A	3.29 ± 0.04	0.17 ± 0.04
**Q58A**	SciW Q58A	2.90 ± 0.11	0.13 ± 0.01
	SciW-TMD Q58A	2.95 ± 0.01	0.15 ± 0.02
	EagT6 Q58A	2.70 ± 0.10	0.11 ± 0.01
	EagT6-TMD Q58A	3.32 ± 0.05	0.22 ± 0.02
**L66A**	SciW L66A	2.91 ± 0.14	0.12 ± 0.03
	SciW-TMD L66A	3.33 ± 0.08	0.14 ± 0.03
	EagT6 L66A	2.89 ± 0.09	0.12 ± 0.01
	EagT6-TMD L66A	3.41 ± 0.01	0.21 ± 0.02
**Q106/2A**	SciW Q106A	2.97 ± 0.02	0.12 ± 0.03
	SciW-TMD Q106A	3.23 ± 0.02	0.17 ± 0.02
	EagT6 Q102A	2.96 ± 0.07	0.15 ± 0.03
	EagT6-TMD Q102A	3.26 ± 0.02	0.16 ± 0.03

Hydrodynamic radius (nm) and polydispersity index (PDI) of Eag wild-type and point variants. Dynamic light scattering (DLS) measurements were performed in DLS buffer (50 mM Tris pH 8.0, 250 mM NaCl, 1 mM BME) at a concentration of 0.5mg/mL at 25 °C. Data represents the average and standard deviation of three individual replicates each measured 10 times each. The refractive index and viscosity of the DLS buffer was calculated using the known values for standard buffer compositions using the Prometheus Panta analysis software.

Supplementary Table 3: NanoDSF thermal and chemical denaturation values and fitting parameters for Eag and Eag:TMD point variants.

Variant	Protein	Tm			GdmCl	
		°C	Δ°C	R^2	C_{50} [M]	R^2
I25/4F	SciW I25F	54.5	25.9	0.950	1.05	0.995
	SciW-TMD I25F	80.4		0.934	2.80	0.992
	EagT6 I24F	53.9	6.4	0.917	0.88	0.995
	EagT6-TMD I24F	60.3		0.983	0.99	0.992
S41A	SciW S41A	54.9	29.0	0.974	1.44	0.974
	SciW-TMD S41A	83.9		0.985	3.20	0.989
	EagT6 S41A	54.0	10.4	0.834	0.84	0.993
	EagT6-TMD S41A	64.4		0.984	1.38	0.992
Q58A	SciW Q58A	54.2	25.3	0.896	1.24	0.993
	SciW-TMDQ58A	79.5		0.991	2.72	0.995
	EagT6 Q58A	52.5	11.2	0.934	1.00	0.997
	EagT6-TMD Q58A	63.7		0.974	1.37	0.994
L66A	SciW L66A	56.8	25.1	0.899	1.21	0.996
	SciW-TMD L66A	81.9		0.972	3.11	0.992
	EagT6 L66A	48.4	4.5	0.599	0.72	0.989
	EagT6-TMD L66A	52.9		0.907	0.64	0.978
Q106/2A	SciW Q106A	55.7	30.3	0.887	1.28	0.997
	SciW-TMD Q106A	86.0		0.918	3.62	0.996
	EagT6 Q102A	45.6	10.4	0.944	0.96	0.995
	EagT6-TMD Q102A	56.0		0.989	1.00	0.994

Thermal denaturation curves were fit to a gaussian distribution and coefficients of determination (R2) values are provided. The Δ°C represents the difference in Tm between the apo and TMD-bound chaperone for each point variant. All proteins were subjected increasing concentrations of guanidine hydrochloride (GdmCl) and the denaturation profiles were fit to a variable slope model with C_{50} [M] and R^2 values provided using the Prometheus NT.48 software and GraphPad Prism.

Supplementary Table 4: Primers for the generation of protein constructs

Expression Construct	Original Construct	F Primer (5' to 3')	R Primer (3' to 5')
**SciW-TMD I24F**	SciW-TMD	CAGCGTCAATTTTTTTATCCTGG	CGATCCGTAAATGTTTCAG
**SciW-TMD S41A**	SciW-TMD	CCTGAATATTGCCCGCGATACGC	CTGGGCGATGTTCGTTCATTG
**SciW-TMD L66A**	SciW-TMD	GAAAAAAAATGCCGGTCAGCAACCGG	ATCAGTGCAATCTGGCGG
**SciW-TMD Q58A**	SciW-TMD	TATTGACCGCTTCATTGCACTGATGAAAAAAAATATCG	TAGGCGGGCAGGTCTTCA
**EagT6-TMD I24F**	EagT6-TMD	GAGCATCAACTTCTTCAAGCTCCCC	TGGTCCTGCCAGGCATCG
**EagT6-TMD S41A**	EagT6-TMD	TTTCGTCATCGCCCGTGACGCCAGCCAGG	CTGGCTTCGCGGGCGG
**EagT6-TMD Q58A**	EagT6-TMD	TGTCGCCCGCGCACTGGAAAACGCC	TAGTCGGCGAACGGCGC
**EagT6-TMD L66A**	EagT6-TMD	CGAGAAGCAAGCGCCCGGCTTCAAG	GCGTTTTCCAGTTGGCGG
**EagT6-TMD Q102A**	EagT6-TMD	GATGCTGCGCTTTGTATTCATCGAGCGCCGCCCGG	AAGTCGCGGCCCTCGCGC

Supplementary Table 5: Strains used in this study.

Organism	Genotype	Description	References
Pseudomonas aeruginosa PAO1	Wild type		[1]
	∆PA4856	*retS* deletion strain	[2]
	∆PA4856 ∆PA0093 ∆PA0092 attB::*lacZ*	*retS, tse6/tsi6* deletion strain	[3]
	∆PA4856 ∆PA0094	*retS, eagT6* deletion strain	[4]
	∆PA4856 PA0094_I24F	*retS* deletion, *eagT6* I24F mutation	This study
	∆PA4856 PA0094_S41A	*retS* deletion, *eagT6* S41A mutation	This study
	∆PA4856 PA0094_Q58A	*retS* deletion, *eagT6* Q58A mutation	This study
	∆PA4856 PA0094_L66A	*retS* deletion, *eagT6* L66A mutation	This study
	∆PA4856 PA0094_Q102A	*retS* deletion, *eagT6* Q102A mutation	This study
E. coli XL-1 Blue	*recA1 endA1 gyrA96 thi-1 hsdR17 supE44 relA1 lac* [F´ *proAB lacI*^{q} ZΔ*M15* Tn*10* (Tet^R)]	Cloning strain	Novagen
E. coli SM10 λpir	*thi thr leu tonA lac Y supE recA*::RP4-2-Tc::Mu	Conjugation strain	BioMedal LifeScience
E. coli BL21 (DE3) CodonPlus	F$^-$ ompT gal dcm lon hsdS$_B$(r$_B^-$ m$_B^-$) λ(DE3) pLysS(cm^R)	Protein expression strain	Novagen
Salmonella Typhimurium SL1344	Wild type		[5]
	SL1344_∆0285	*sciW* deletion, the gene is STM_0290 in LT2	[5]
	SL1344_∆0266	*clpV* deletion, the gene is STM_0272 in LT2	[5]

Supplementary Table 6: Plasmids used in this study

Plasmid	Relevant features	Reference
pETDuet-1:: SL1344_0286_1-59-His_6:: SL1344_0285-VSV-G	Co-expression vector for C-terminal His_6 tagged Rhs1 TMD and C-terminal VSV-G tagged SciW	[6]
pETDuet-1::PA0093_1-61-His_6::PA0094-VSV-G	Co-expression vector for C-terminal His_6 tagged Tse6 TMD1 and C-terminal VSV-G tagged EagT6	[6]
pRSETA::SL1344_0285	Expression vector for SciW from *Salmonella* Typhimurium (for crystallization)	[6]
pET29b::SL1344_0285-VSVG	Expression vector for SciW	This study
pET28a::PA0094	Expression vector for EagT6	
pET29b::PA0094-VSV-G	Expression vector for C-terminal VSV-G tagged EagT6 from *P. aeruginosa*	[6]
pETDuet-1::SL1344_0286_1-59-His_6:: SL1344_0285-VSV-G I24F	*sciW*_TMD I24F mutation construct	This study
pETDuet-1::SL1344_0286_1-59-His_6:: SL1344_0285-VSV-G S41A	*sciW*_TMD S41A mutation construct	This study
pETDuet-1::SL1344_0286_1-59-His_6:: SL1344_0285-VSV-G Q58A	*sciW*_TMD Q58A mutation construct	This study
pETDuet-1::SL1344_0286_1-59-His_6:: SL1344_0285-VSV-G L66A	*sciW*_TMD L66A mutation construct	This study
pETDuet-1::SL1344_0286_1-59-His_6:: SL1344_0285-VSV-G Q106A	*sciW*_TMD Q106A mutation construct	This study
pETDuet-1::PAA0093_1-61-His6::PA0094-VSV G I24F	*eagT6*_TMD I24F mutation construct	This study
pETDuet-1::PAA0093_1-61-His6::PA0094-VSV G S41A	*eagT6*_TMD S41A mutation construct	This study
pETDuet-1::PAA0093_1-61-His6::PA0094-VSV G L66A	*eagT6*_TMD L66A mutation construct	This study
pETDuet-1::PAA0093_1-61-His6::PA0094-VSV G Q58A	*eagT6*_TMD Q58A mutation construct	This study
pETDuet-1::PAA0093_1-61-His6::PA0094-VSV G Q102A	*eagT6*_TMD Q102A mutation construct	This study
pET29b::SL1344_0285-VSVG I24F	*sciW* I24F mutation construct	This study
pET29b::SL1344_0285-VSVG S41A	*sciW* S41A mutation construct	This study
pET29b::SL1344_0285-VSVG Q58A	*sciW* Q58A mutation construct	This study
pET29b::SL1344_0285-VSVG L66A	*sciW* L66A mutation construct	This study
pETDuet-1::SL1344_0285-VSVG Q106A	*sciW* Q106A mutation construct	This study
pET29b::PA0094-VSV-G I24F	*eagT6* I24F mutation construct	This study
pET29b::PA0094-VSV-G S41A	*eagT6* S41A mutation construct	This study
pET29b::PA0094-VSV-G Q58A	*eagT6* Q58A mutation construct	This study
pET29b::PA0094-VSV-G L66A	*eagT6* L66A mutation construct	This study
pET29b::PA0094-VSV-G Q102A	*eagT6* Q102A mutation construct	This study
pEXG2	Allelic replacement vector containing *sacB,* Gm^R	[7]
pEXG2::PA0094_I24F	*eagT6* I24F mutation construct	This study
pEXG2::PA0094_S41A	*eagT6* S41A mutation construct	This study
pEXG2::PA0094_Q58A	*eagT6* Q58A mutation construct	This study
pEXG2::PA0094_L66A	*eagT6* L66A mutation construct	This study
pEXG2::PA0094_Q102A	*eagT6* Q102 mutation construct	This study

Supplementary Table 7: Primers for the generation of mutant strains

Name	Sequence
**Eag_pointmutant_P1_XbaI**	TCAATCAGTATCTAGATTCCAGCGCCAGGCTGGCC
**Eag_pointmutant_P4_KpnI**	TGTTAAGCTAGGTACCGAACGGCCACAGCGGATGG
**EagT6_I24F_check**	CTGGCGGGGAGCTTGAAGAA
**EagT6_I24F_P2**	GGGAGCTTGAAGAAGTTGATGCTC
**EagT6_I24F_P3**	AGCATCAACTTCTTCAAGCTCCCC
**EagT6_L66A_check**	CAGCTTGAAGCCGGGGGC
**EagT6_L66A_P2**	TTGAAGCCGGGGGCTTGCTTC
**EagT6_L66A_P3**	AGCAAGCCCCCGGCTTCAAG
**EagT6_Q58A_check**	GACTATGTCGCCCGCGCC
**EagT6_Q58A_P2**	TTTCCAGGGCGCGGGCG
**EagT6_Q58A_P3**	GCCCGCGCCCTGGAAAAC
**EagT6_Q102A_check**	GGCGCTCGATGAATACCGC
**EagT6_Q102A_P2**	ATGAATACCGCGCGCAGCATC
**EagT6_Q102A_P3**	TGCTGCGCGCGGTATTCATCG
**EagT6_S41A_check**	GAAGCCAGTTTCGTCATCGCC
**EagT6_S41A_P2**	CGTCACGGGCGATGACGAAAC
**EagT6_S41A_P3**	CGTCATCGCCCGTGACGC

Supplemental References

1 Stover, C. K. *et al.* Complete genome sequence of Pseudomonas aeruginosa PAO1, an opportunistic pathogen. *Nature* **406**, 959-964 (2000). https://doi.org/10.1038/35023079

2 Ahmad, S. *et al.* An interbacterial toxin inhibits target cell growth by synthesizing (p)ppApp. *Nature* (2019). https://doi.org/10.1038/s41586-019-1735-9

3 Whitney, J. C. *et al.* Genetically distinct pathways guide effector export through the type VI secretion system. *Mol Microbiol* **92**, 529-542 (2014). https://doi.org/10.1111/mmi.12571

4 Whitney, J. C. *et al.* An interbacterial NAD(P)(+) glycohydrolase toxin requires elongation factor Tu for delivery to target cells. *Cell* **163**, 607-619 (2015). https://doi.org/10.1016/j.cell.2015.09.027

5 Mulder, D. T., Cooper, C. A. & Coombes, B. K. Type VI secretion system-associated gene clusters contribute to pathogenesis of Salmonella enterica serovar typhimurium. *Infection and Immunity* **80**, 1996-2007 (2012). https://doi.org/10.1128/IAI.06205-11

6 Ahmad, S. *et al.* Structural basis for effector transmembrane domain recognition by type VI secretion system chaperones. *Elife* **9** (2020). https://doi.org/10.7554/eLife.62816

7 Rietsch, A., Vallet-Gely, I., Dove, S. L. & Mekalanos, J. J. ExsE, a secreted regulator of type III secretion genes in Pseudomonas aeruginosa. *Proc Natl Acad Sci U S A* **102**, 8006-8011 (2005). https://doi.org/10.1073/pnas.0503005102
